# Transcription Factor Functional Protein-Protein Interactions in Plant Defense Responses

**DOI:** 10.3390/proteomes2010085

**Published:** 2014-03-04

**Authors:** Murilo S. Alves, Silvana P. Dadalto, Amanda B. Gonçalves, Gilza B. de Souza, Vanessa A. Barros, Luciano G. Fietto

**Affiliations:** Department of Biochemistry and Molecular Biology, Federal University of Viçosa, Viçosa, Minas Gerais 36570000, Brazil; E-Mails: murilobqi@yahoo.com.br (M.S.A.); sil_dadalto@yahoo.com.br (S.P.D.); amandabonoto@gmail.com (A.B.G.); gilzab18@gmail.com (G.B.S.); vanessa.barros.ufv@gmail.com (V.A.B.)

**Keywords:** biotic stress, transcription factor, signaling cascades

## Abstract

Responses to biotic stress in plants lead to dramatic reprogramming of gene expression, favoring stress responses at the expense of normal cellular functions. Transcription factors are master regulators of gene expression at the transcriptional level, and controlling the activity of these factors alters the transcriptome of the plant, leading to metabolic and phenotypic changes in response to stress. The functional analysis of interactions between transcription factors and other proteins is very important for elucidating the role of these transcriptional regulators in different signaling cascades. In this review, we present an overview of protein-protein interactions for the six major families of transcription factors involved in plant defense: basic leucine zipper containing domain proteins (bZIP), amino-acid sequence WRKYGQK (WRKY), myelocytomatosis related proteins (MYC), myeloblastosis related proteins (MYB), APETALA2/ ETHYLENE-RESPONSIVE ELEMENT BINDING FACTORS (AP2/EREBP) and no apical meristem (NAM), Arabidopsis transcription activation factor (ATAF), and cup-shaped cotyledon (CUC) (NAC). We describe the interaction partners of these transcription factors as molecular responses during pathogen attack and the key components of signal transduction pathways that take place during plant defense responses. These interactions determine the activation or repression of response pathways and are crucial to understanding the regulatory networks that modulate plant defense responses.

## 1. Introduction

The growth and development of plants are constantly affected by various environmental stresses, and among the most important biotic stresses are those caused by viruses, bacteria, fungi and nematodes [1]. Plants withstand pathogenic attacks by activating a large variety of defense mechanisms, including the hypersensitive response (HR), the induction of genes that encode pathogen-related proteins (PR), the production of antimicrobial compounds called phytoalexins, the generation of reactive oxygen species (ROS), and enhancement of the cell wall [1]. The response mechanisms of these complexes are finely regulated by a large number of genes that encode regulatory proteins. A typical example of a regulatory protein is a transcription factor [2]. Transcription factors are primordial proteins that respond to stress, altering the expression of a cascade of defense genes [2]. Many of these transcription factors are co-induced in response to different stressors suggesting the existence of complex interaction [2].

Transcription factors are defined as transcriptional regulators that function by binding to specific *cis*-regulatory elements present in the promoters of target genes [3]. Transcriptional regulation plays a central role in the control of gene expression in plants, with approximately 2,000 genes predicted to be transcription factors in *Arabidopsis thaliana* [4].

In plants, the main families of transcription factors responsible for the regulation of genes responsive to pathogens are categorized into the following families: a family of proteins that contain either one or two 60-amino-acid regions that contain the amino-acid sequence WRKYGQK (WRKY); APETALA2/ETHYLENE-RESPONSIVE ELEMENT BINDING FACTORS family (AP2/ERF); basic leucine zipper containing domain proteins (bZIP); myelocytomatosis related proteins (MYC); myeloblastosis related proteins (MYB) and, more recently, the no apical meristem (NAM), Arabidopsis transcription activation factor (ATAF), and cup-shaped cotyledon (CUC), or also termed NAC family [1,5]. Each transcription factor family has a specific binding domain such as bZIP, zinc finger, or helix turn helix. These domains bind to DNA *cis*-elements associated with the response to a specific environmental stress set, and the differences between these domains are key features that distinguish one family from another [1,5].

Modulating the function of transcription factors through interactions with regulatory proteins is a crucial process in the activation or repression of signal transduction pathways [1,5]. Processes such as effector-triggered immunity (ETI), which results in a rapid process of programmed cell death known as the hypersensitive response (HR), and pathogen-associated molecular pattern (PAMP)-triggered immunity (PTI), which results in the prevention of infection by the pathogen, are finely regulated by the interactions between different proteins with transcription factors [6,7,8]. Several proteins have been reported to modulate the function of various plant transcription factors, such as the NON-EXPRESSER OF PATHOGEN-RELATED (PR) GENES (NPR1) protein, which binds to the TGACGTCA *cis*-element-binding protein (TGA) factor of the basic leucine zipper domain (bZIP) family during the activation of salicylic acid (SA) signaling [6,7,8], and the MITOGEN-ACTIVATED PROTEIN (MAP) kinases, which also have a proven role in regulating WRKY family *trans*-acting factors [9]. In this paper, we discuss the current understanding of the interactions between transcription factors and several regulatory proteins that modulate the activities of these *trans*-acting factors by various mechanisms, such as inactivation, subcellular localization, degradation and post-translational modification, and the manner in which these interactions affect signal transduction pathways in plant defenses against environmental challenges.

## 2. bZIP Family

The family of transcription factors containing the bZIP domain is one of the largest families of transcriptional factors in eukaryotes. In plants, these factors regulate genes in response to abiotic stress, seed maturation, floral development and defense against pathogens [10]. Jakoby and collaborators classified bZIP proteins from *Arabidopsis* (AtbZIPs) into 10 distinct groups: A, B, C, D, E, F, G, H, I and S.

**Figure 1:**
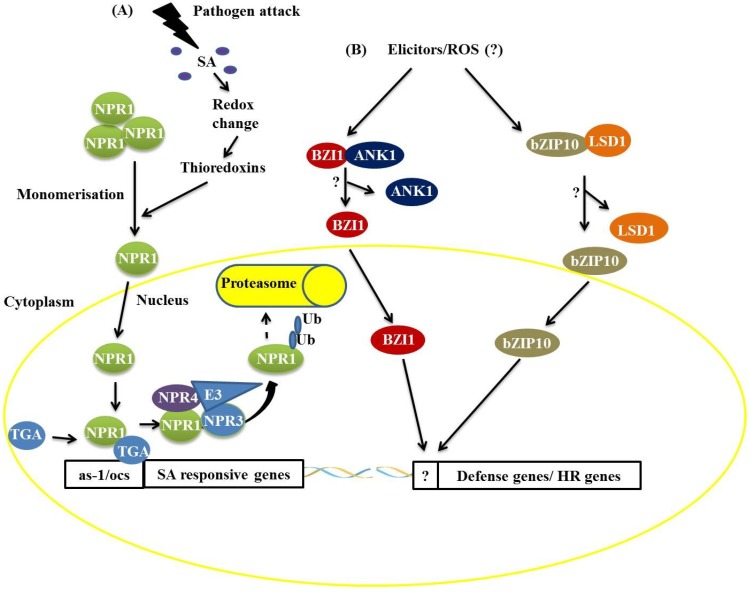
Two distinct mechanisms of basic leucine zipper containing domain proteins (bZIP) protein actions during plant defense responses. (**A**) The attack of a biotrophic pathogen triggers a signaling pathway mediated by salicylic acid resulting in the dissociation of the non-expresser of pathogen-related (PR) (NPR1) protein, which translocates to the nucleus and activates the expression of SA-responsive genes by interaction with the TGACGTCA *cis*-element-binding protein (TGA) bZIP *trans*-acting factors. The NPR1 protein is ubiquitinated and targeted for degradation by the 26S proteasome complex; (**B**) Recognition of elicitors after pathogen attack promotes the dissociation of the BZI1/ANK1 and AtbZIP10/LSD1 complexes, favoring the positive transcriptional regulation of hypersensitive response (HR)- and basal defense-related genes.

In the literature, specific interactions of bZIP proteins with other proteins that regulate the bZIP protein’s activity, subcellular localization and function during defense processes against pathogens have been reported [10,11]. Acting as key regulators of signaling mediated by SA, the TGA proteins, members of Group D of the *Arabidopsis* bZIP proteins, comprise a class of bZIP proteins that are linked with responses to biotic stress [10]. A major development in the study of the functional interactions of TGA members during pathogen responses has been the discovery of interactions with members of the ankyrin repeat protein family, specifically NON-EXPRESSER OF PATHOGEN-RELATED (PR) GENES (NPR1), which are key components in the defense signaling pathway mediated by SA [6,7,8]. Under normal conditions, most NPR1 is retained in the cytoplasm as an oligomer via intermolecular disulfide bonds (Figure 1) [6,12]. Under pathogen attack, SA is synthesized and induces changes in the cellular redox state [6,7,8,12], promoting the monomerization of NPR1 through the activity of the THIOREDOXINS H3 and H5 (TRX-H3/H5). In SA-induced cells, monomeric NPR1 translocates into the nucleus *via* the nuclear pore complex (NPC) [6,7,8,12], and the NPR1 monomers interact with members of the TGA family (bZIP) and bind to SA-responsive gene promoters (Figure 1). During this process, NPR1 is phosphorylated and then ubiquitinated by an E3 ubiquitin ligase that has a high affinity for phosphorylated NPR1, thus targeting NPR1 for degradation by the proteasome complex. This process starts in the nucleus and ends in the cytosol (Figure 1) [6,7,8,12]. NPR3 and NPR4, protein homologs of NPR1, act as receptors of SA in this process, binding to this molecule with different affinities. NPR3 and NPR4 serve as Cullin 3, E3 ubiquitin ligase adapters, that mediate the ubiquitination (Ub) and degradation of NPR1 and are regulated by SA (Figure 1) [6,7,8,12]. The *Arabidopsis* double mutants, *npr3 npr4*, accumulate high levels of NPR1 and are insensitive to the induction of systemic acquired resistance [6].

Studies have also demonstrated that 17 CC-type glutaredoxins interact with TGA2 [13]. It has been proposed that this interaction between CC-type glutaredoxins and TGA proteins plays a role not only in defense against pathogens but also in processes involved in plant development [13]. WRKY proteins also interact with TGA proteins [14]. In tobacco, the NtWRKY12 protein interacts *in vitro* and *in vivo* with TGA proteins [14].

In addition to the TGA proteins, it has been demonstrated that AtbZIP10 interacts with LESIONS SIMULATING DISEASE RESISTANCE 1 (LSD1), a protein with a zinc finger domain, *in vivo* (Figure 1) [15,16]. LSD1 is a negative regulator of cell death and protects plant cells from oxidative stress [16]. The interaction between LSD1 and AtbZIP10 occurs in the cytoplasm, resulting in the partial retention of AtbZIP10 (Figure 1) [16]. AtbZIP10 positively regulates basal defense responses and cell death induced by reactive oxygen species (ROS), and these activities are antagonized by LSD1 [16]. Studies have also shown that a protein related to NPR1, an ANKYRIN-REPEAT PROTEIN (ANK1), interacts with a bZIP protein known as BZI1 (Figure 1) [17]. BZI1 has a DNA-binding domain and a D1 domain that is apparently essential for auxin signaling and defense against pathogens [17]. The molecular characterization of ANK1 has demonstrated that this protein is unable to bind to DNA and modulate gene transcription [17]. ANK1 is preferentially localized in the cytosol, and its transcription is negatively regulated under pathogen attack [17]. These features have led to the conclusion that ANK1 is involved in the modulation of auxin signaling and defense against pathogens in a manner dependent on its interaction with members of the bZIP family, such as BZI1 [17].

## 3. AP2/ERF Family

APETALA2/ETHYLENE-RESPONSIVE ELEMENT BINDING FACTORS (AP2/ERF) proteins belong to a family of plant transcription factors that exhibit the AP2/ERF domain necessary for specific binding to DNA and that can be subdivided into four subfamilies defined by Sakuma *et al*. [18]: AP2, DEHYDRATION-RESPONSIVE ELEMENT-BINDING (DREB), ERF and RELATED TO ABI3/VPI (RAV). The subfamily AP2 contains two AP2 domains, AP2/ERF, separated by a linker containing 25 amino acids. While members of the subfamily RAV have, in addition to the AP2/ERF domain, another DNA-binding domain known as B3, members of the subfamilies DREB and ERF contain only one AP2/ERF domain.

**Figure 2:**
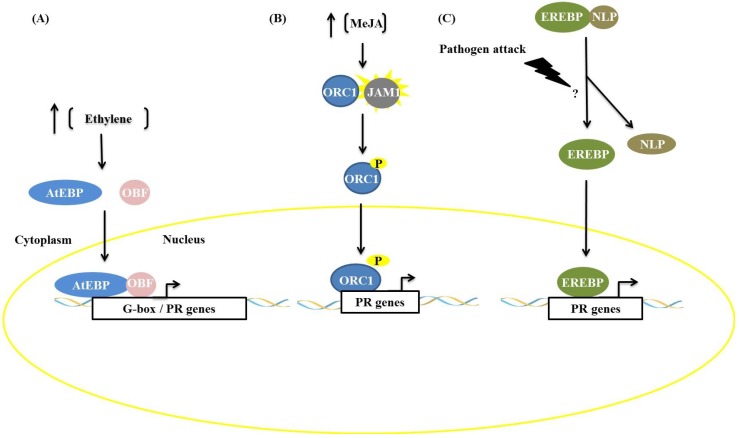
Types of interactions among APETALA2/ETHYLENE-RESPONSIVE ELEMENT BINDING FACTORS (AP2/ERF) factors and other proteins in response to biotic stress. (**A**) Association with other transcription factors: the protein AtEBP binds to OCTOPINE SYNTHASE (ocs) ELEMENTS BINDING FACTOR (OBF) protein, which is a bZIP protein, resulting in increased transcription of PR genes; (**B**) Phosphorylation: the AP2/ERF factor octadecanoid-responsive-*Catharanthus*-APETALA2-domain protein (ORC1) is phosphorylated by kinase JAM1 and promotes expression of genes related to nicotine synthesis; (**C**) Dissociation: after ethylene induction or pathogen infection, the protein EREBP dissociates from NLP protein. This dissociation results in the translocation of EREBP to the nucleus and leads to expression of PR genes.

AP2/ERF transcription factors and other factors frequently act synergistically, increasing the expression of genes related to plant defense, as reported by Singh and Buttner [19]. The AtEBP protein (*Arabidopsis* ethylene binding protein), during activation of the defense pathway mediated by ethylene, recognizes the *cis*-element GCC-box and interacts with a bZIP family protein, OCTOPINE SYNTHASE (ocs) ELEMENTS BINDING FACTOR (OBF), that is able to recognize the G-box (CACGTG) (Figure 2). This interaction increases the expression of PR genes that contain both *cis*-elements. Similarly, in tobacco, the protein TOBACCO STRESS-INDUCED 1 (Tsi1) recruits the zinc-finger-containing Tsi1-INTERACTING PROTEIN1 (TSIP), an interaction demonstrated by two-hybrid assays, Western blotting and co-immunoprecipitation. This interaction results in increased tolerance to *Pseudomonas tabaci*, a hemibiotrophic plant pathogen, and transcription of the genes PATHOGENESIS RELATED PROTEIN 4 (PR4), SYSTEMIC ACQUIRED RESISTANCE PROTEIN 8.2 (SAR8.2) and LIPID TRANSFER PROTEIN (LTP), which are stress-related [19].

Other interactions can result in the phosphorylation of AP2/ERF proteins. When the ethylene signaling pathway is induced, phosphorylation can occur via MAPK kinases, such as the pair OsEREBP1/BWMK1 in rice [20] and TaERF1/TaMAPK1 in wheat [21], or by Ser/Thr kinases, such as the *Pseudomonas tomato* resistance-interacting4 (Pti4) and *Pseudomonas tomato* resistance (Pto) kinase of tomato [22]. In tobacco, the transcription factor octadecanoid-responsive-*Catharanthus*-APETALA2-domain protein (ORC1) can be phosphorylated by MAP kinases or other kinases [23]. In all the examples mentioned, phosphorylation results in increased activity of the transcription factor ORC1. Another example of an interaction that regulates the activity of AP2/ERFs is that of EREBP2 with the protein NITRILASE-LIKE PROTEIN (NLP), proposed by Xu *et al*. [24], where NLP proteins associate with EREBP proteins and retain these factors in the cytoplasm. Contact with elicitors result in a dissociation process, and the factor EREBP is translocated into the nucleus where it promotes the expression of PR genes (Figure 2C) [24].

## 4. MYB Family

During a pathogenic infection, the expression of myeloblastosis related (MYB) family of transcription factors is diverse and present in all eukaryotes. This family has a variable number of MYB domains, which influence the capacity to bind to DNA [25]. The *N*-terminal region of the protein contains the DNA-binding domain and is highly conserved. The C-terminal region may contain a domain necessary for activation or transcriptional repression. Based on this structure, these proteins are divided into four classes: 1R, R2R3, 3R and 4R [26], and the R2R3-MYB class is divided into 22 subgroups [27].

**Figure 3:**
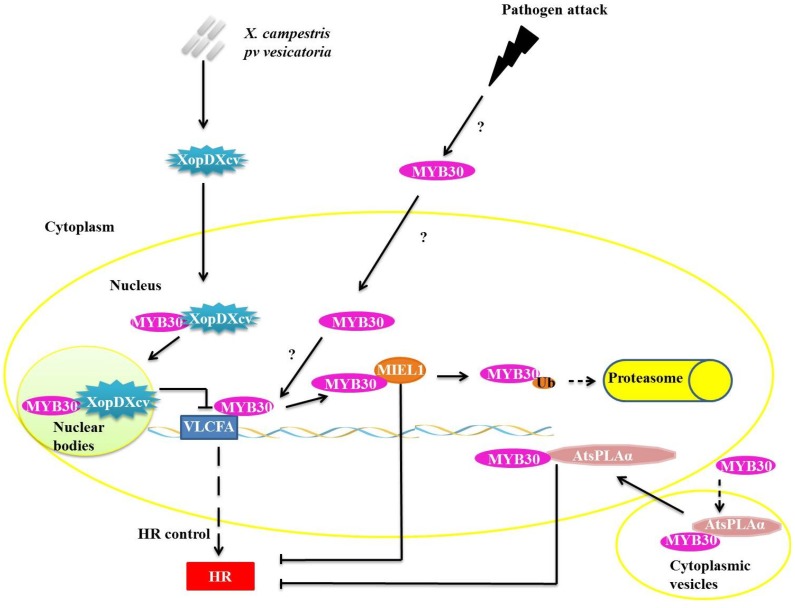
Repression mechanisms of myeloblastosis related proteins (MYB)30 function during pathogen attack. XopDXcv interacts with MYB30 in plant cell nucleus, retaining MYB30 in nuclear bodies and preventing the transcription of the very long chain fatty acid biosynthesis genes (VLCFA) genes. Ubiquitin ligase E3 MYB30-INTERACTING E3 LIGASE1 (E3 MIEL1) interacts with MYB30 in the nucleus and promotes its ubiquitination and consequent degradation by the 26S proteasome complex (UPS26). AtsPLAα binds with MYB30 and they translocate from the cytoplasmic vesicles into the nucleus, but the interaction of AtsPLAα with target DNA is prevented.

The proteins of the R2R3-MYB class are plant-specific and are involved in the following processes: primary and secondary metabolism, cell destination and identity, development and responses to abiotic and biotic stress [26]. Previous studies have verified that *Arabidopsis* AtMYB30 over-expression accelerates and intensifies the hypersensitivity response (HR) after attack from avirulent strains of *Pseudomonas syringae*, suggesting that it acts as a positive regulator of cell death in response to the attack of pathogenic bacteria [27]. MYB30 targets very long chain fatty acid biosynthesis genes (VLCFA) during pathogen infection (Figure 3). VLCFAs and their derivatives are likely involved in the establishment or control of HR [28]. To control the concentration of MYB30, the enzyme ubiquitin ligase E3 MYB30-INTERACTING E3 LIGASE1 (MIEL1) interacts specifically with MYB30 in the plant cell nucleus (Figure 3). MIEL1 ubiquitinates MYB30, targeting it for degradation in the 26S proteasome. The *Arabidopsis* mutant *miel1* presents increased HR and resistance to avirulent bacteria*.* The expression of MIEL1 is inhibited during infiltration of avirulent *P. syringae*, enabling the accumulation of the MYB30 required to promote HR and, consequently, restricting the propagation of the bacteria to other regions of the tissue [29].

In one known mechanism of suppression of plant defense responses, XopDXcv, one of the Type III effectors of *Xanthomonas campestris pv. vesicatoria* specifically interacts with the HLH domain of MYB30 and promotes its localization to nuclear bodies (Figure 3). The localization of MYB30 into the nuclear bodies prevents the activation of genes related to synthesis of VLCFA, preventing the appropriate activation of plant defense pathways [30]. The reprogramming of the host’s transcription by XopD represents a virulence strategy that allows for the establishment of infections by the *Xanthomonas* species [30].

In plants, the PHOSPHOLIPASE A2S (AtsPLAα) is related to growth, development, stress responses and defense signaling. AtsPLAα is a negative regulator of HR and defense responses in *Arabidopsis* and is mediated specifically by AtMYB30 localized in cytoplasmic vesicles, preventing the transcription of genes normally mediated by AtMYB30 (Figure 3) [31].

**Figure 4:**
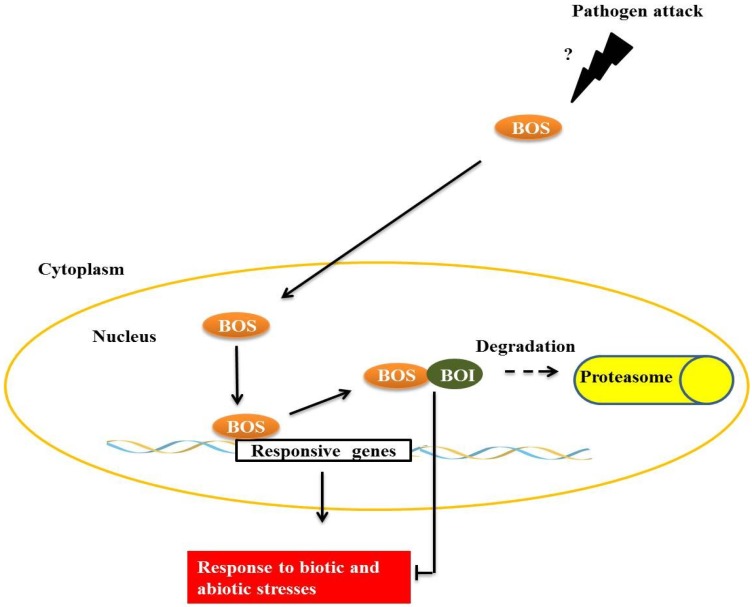
The transcription factor, *BOTRYTIS* SUSCEPTIBLE (BOS), interacts with E3 *BOTRYTIS* SUSCEPTIBLE1 INTERACTOR (BOI) in the plant cell nucleus. E3 BOI promotes BOS ubiquitination and the consequent degradation by the 26S proteasome complex, restricting the biotic and abiotic stress responses mediated by BOS.

*BOTRYTIS* SUSCEPTIBLE 1 (BOS1), a transcription factor of the R2R3MYB subgroup termed AtMYB108/BOS1, is necessary for responses to biotic and abiotic stresses in *Arabidopsis*. Mutants present a higher susceptibility to necrotic lesions and also have less tolerance to water deficits, salinity and oxidative stress when compared with wild type [32]. BOS physically interacts with *BOTRYTIS* SUSCEPTIBLE1 INTERACTOR (BOI) in plant cell nuclei through the central preserved domain dominated WRD, a region that is important in forming the coiled-coil structure that is often important for protein-protein interactions [32] (Figure 4). BOI is a one RING E3 ligase able to ubiquitinate the protein R2R3MYB *in vitro*, and possibly *in vivo*, leading to subsequent degradation by the proteasome. Plants with BOI silenced by RNAi are much more susceptible to *Botrytis cinerea* and less tolerant to salinity [33], similar to observations made of the *bos1* mutant [32]. Curiously, RNAi-BOI plants expressing 35S:BOS1-GUS are more resistant to fungi than wild-type plants, suggesting that BOS1 is a direct target of BOI. Expression of BOI is induced by SA and 1-aminocyclopropane-1-carboxylic acid (ACC), which is a precursor compound of the ethylene biosynthesis pathway, but is inhibited by methyl jasmonate (MeJA) and gibberellins (GAs), presenting evidence for the complex regulation that is responsible for maintaining a normal level of BOI in wild plants. However, the occurrence of *B. cinerea* infections is known to be increased by the accumulation of SA, ET, MeJA and abscisic acid in wild plants [33].

## 5. MYC Family

The myelocytomatosis related family (MYC) represents a subfamily of transcription factors that contain a basic-Helix-Loop-Helix (bHLH) domain, is present in all eukaryotes, and is characterized by having a basic DNA-binding region in the *N*-terminal region and, in the *C*-terminal region, hydrophobic residues that form two alpha helices separated by a loop, which determine the protein’s dimerization capacity. The bHLH domain is characteristic of a large family of bHLH transcription factors to which MYC belongs [34].

MYC transcription factors are key transcriptional regulators in the expression of jasmonate (JA)-responsive genes, positively regulating wound resistance genes and acting as negative regulators during the expression of pathogen defense genes [1,35]. Under pathogen attack and herbivory, plants produce JA conjugated with isoleucine (JA-Ileu, a JA bioactive form), which is recognized and bound by its receptor CORONATINE INSENSITIVE-1 (COI1). The COI1 protein is an F-box protein that associates with the cullin, SKP1 and RBX1 proteins, together forming the SCF^COI1^ complex. The presence of JA-Ileu and its surrounding sequence allows the protein to bind to COI1, leading to a switch in the jasmonate-zinc-finger protein expression in inflorescence meristem. The JASMONATE-ZIM-DOMAIN (JAZ) proteins and their binding partners lead to JAZ unbinding from MYC. JAZ interacts, by means of its Jas domain, with the SCF^COI1^ complex. JAZ is then ubiquitinated by the complex and sequentially degraded by the 26S proteasome [35,36,37,38,39,40]. Thus, in the presence of JA-Ileu, JAZ quickly undergoes proteolysis, promoting the release and activation of MYC. MYC activation also results in the expression of other transcription factors, such as MYBs and WRKYs, which are important in stress defense [40]. In addition, MYC activates the transcription of the JAZ protein, leading to a basal level restoration of JA [37].

**Figure 5:**
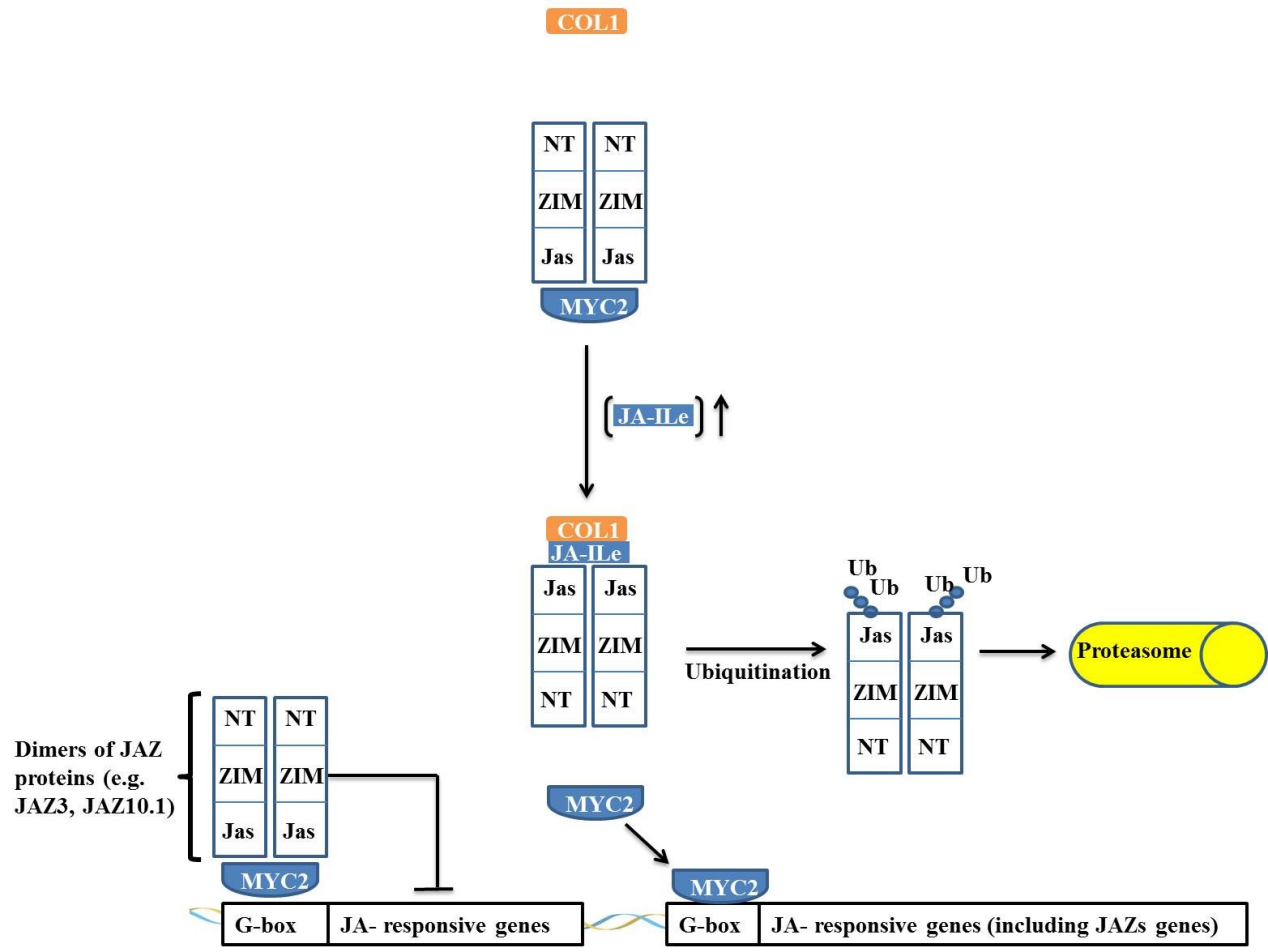
Regulation of jasmonate-responsive gene expression by MYC2 and JAZ proteins. In absence of JA-Ileu, JAZ protein interacts through its *N*-terminal domain with MYC2, causing the transcription factor to remain inactive. When the JA-Ileu level increases, JA-Ileu binds to Jas domain of JAZ protein and promotes interaction of JAZ protein with COI1 leading to the formation of the SCF^COI1^ complex. The SCF^COI1^ complex causes ubiquitination of JAZ protein in its Jas domain and the protein is degraded by the 26S proteasome complex. MYC2 is released and promotes transcription of target genes.

JAZ proteins are composed of a family of 12 proteins that contain a centrally located ZIM domain on the C-terminal side of the JASMONATE-ASSOCIATED (Jas) domain and in the N-terminal region. JAZ proteins act as suppressors of the JA response, and the majority of JAZ proteins (such as JAZ3 and JAZ10.1), in the absence of JA-Ileu, have the ability to interact with MYC and negatively regulate its activity (Figure 5) [36].

JAZ proteins interact with MYC2 through their N-terminal portion, and when the Jas domain is truncated, the JAZ protein is not degraded, remaining irreversibly bound to MYC2 and acting as a dominant-negative repressor. This effect indicates that JAZ proteins do not require a Jas domain to interact with MYC2 and that repression occurs through an interaction of the JAZ *N*-terminal domain with MYC2 (Figure 5) [37]. This interaction and regulation model of MYC is not applicable to all JAZ proteins because the interaction of the JAZ3 protein with MYC2 has been described as occurring *via* a different mechanism. A Jas domain deletion in JAZ3 renders this protein unable to interact with MYC2, and it has been demonstrated that the Jas domain itself is sufficient for the interaction of JAZ3 with MYC2 [38]. Thus, it is proposed that JAZ3 interacts by binding as a dimer through the Jas domain to MYC2, suppressing its action (Figure 5). An interesting observation is that MYC2 is irreversibly inactivated by the truncated protein that is derived from a deletion in the *C*-terminal region of JAZ3. It has been proposed that this interaction occurs through heterodimerization with another JAZ protein through its *N*-terminal domain, which, in turn, binds irreversibly to MYC2, thus acting as a dominant-negative repressor [37].

In *Arabidopsis*, MYC2 is able to interact with all 12 of the JAZ proteins, whereas MYC3 demonstrates a strong interaction with only eight of these proteins (JAZ1, JAZ2, JAZ5, JAZ6, JAZ8, JAZ9, JAZ10 and JAZ11) [39] and MYC4 interacts with only JAZ1, JAZ3 and JAZ9 [1]. All of the mechanisms of interaction are similar to that described for MYC2 [1,39].

## 6. WRKY Family

The defining feature of the WRKY transcription factors is their DNA-binding domain, a highly conserved region of 60 amino acids. In this region, there is a nearly invariable sequence, WRKYGQK, and the *N*-terminal portion of the protein is followed by a zinc finger motif, Cx4-HxC 5Cx22-23HxH or Cx7Cx23 [41].

WRKY factors are divided into three groups based on the number of WRKY domains in the protein and the structure of their zinc fingers [42]. Group II genes have been subdivided into IIa, IIb, IIc, IId and lIe on the basis of their amino acid sequence. Another division uses phylogenetic data and suggests that the WRKY family in higher plants should be divided into groups I, IIa + IIb, IIc, IId + IIe, and III [43,44]. WRKY transcription factors generally bind to a conserved sequence of DNA known as the W-box, (T) (T) TGAC (C/T) [42].

WRKY proteins are implicated in various molecular events in plants, such as seed development, senescence, dormancy and germination, and abiotic and biotic stresses among others [41]. A large number of members of the WRKY family are related to pathogen infection and thus are important factors for plant immunity. Some WRKY protein partners have already been identified, and the interactions between WRKY and its binding partners may play roles in signaling, transcription, chromatin remodeling, and other cellular processes [45].

**Figure 6:**
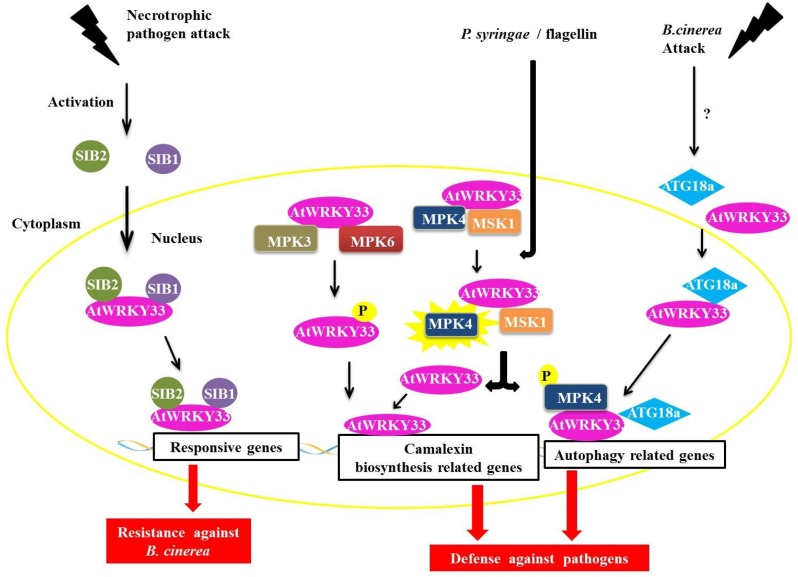
Overview of AtWRKY33 interactions during biotic stress responses. During an attack by a necrotrophic pathogen, AtWRKY33 interacts with the proteins SIGMA FACTOR-INTERACTING PROTEIN 1 (SIB1) and SIGMA FACTOR-INTERACTING PROTEIN 2 (SIB2) in the nucleus. These interaction leads to transcription of genes responsive to the pathogen, causing an increased resistance in the plant (in this case against *B. cinerea*, a necrotrophic fungus). In a second interaction, AtWRKY33 can be phosphorylated by two MITOGEN-ACTIVATED PROTEIN (MAP) kinases, MITOGEN-ACTIVATED PROTEIN KINASE 3 (MPK3) and MPK6. This interaction leads to an increase in the transcription of related genes of camalexin biosynthesis, which is an important pathway utilized by the plant defense against pathogens. Another interaction leads to increased transcription of camalexin related genes. After induction by *Pseudomonas syringae* or flagellin, the protein MPK4 is activated and phosphorylates its substrate, the MAP KINASE SUBSTRATE1 (MSK1) protein. Phosphorylation of MSK1 releases AtWRKY33 of protein complex allowing the protein to exert its role as a transcriptional activator of plant defense genes. Finally, during attack of fungus *B. cinerea*, AtWRKY33 interacts with ATG18a in the nucleus. ATG18a is an important protein of the autophagy pathway in *Arabidopsis*, and its interaction with AtWRKY33 along with the activation of the autophagy pathway is important for signaling the response of plant defense against necrotrophic pathogens.

The AtWRKY33 protein in *Arabidopsis* plays an important role during infection by necrotrophic pathogens and is a part of the group I WRKY family [46]. AtWRKY33 interacts with the proteins SIGMA FACTOR-INTERACTING PROTEIN 1 and 2 (SIB1 and SIB2) (Figure 6) [47]. The SIB1 and SIB2 proteins are classified as VQ proteins because they have the conserved FXXXVQXLTG or VQ motif [48,49,50]. The proteins AtWRKY33, SIB1 and SIB2 are induced by the necrotrophic fungus *Botrytis cinerea*, which is also coordinately regulated during infection with this pathogen. Through the BiFC assay, we determined that the interaction between SIB1 and SIB2 occured in the nucleus of the plant cell (Figure 6). Tests with deletion mutants *sib1* and *sib2* showed a decrease in plant resistance to *B. cinerea*, whereas in plants, over-expressing the mutant protein SIB1 led to increased resistance to the fungus. These experiments indicate a positive role for these two proteins as AtWRKY33 activators but that they are not essential in defense-mediated AtWRKY33 in plants [47].

Other interaction partners have been described for the AtWRKY33 protein, including one MAPK (MITOGEN-ACTIVATED PROTEIN KINASE) or MPK4 and its substrate, a VQ protein called MAP KINASE SUBSTRATE1 (MSK1) (Figure 6). In addition to AtWRKY33, the AtWRKY25 protein is also capable of interacting with MPK4 and MSK1 [48,50]. It has been proposed that interactions with AtWRKY25 in the absence of the pathogen are in the form of a nuclear-localized complex between MPK4, MKS1 and AtWRKY33. After induction by either *Pseudomonas syringae* or flagellin (a protein found in bacterial flagella), the MPK4 protein is activated and phosphorylates its substrate, MSK1. MSK1 phosphorylation releases the AtWRKY33 complex, allowing AtWRKY33 to bind to the promoter region of some genes, including the phytoalexin deficient3 (PAD3) promoter, which encodes an enzyme that participates in the synthesis of the antimicrobial compound camalexin, a type of phytoalexin that plays an important role in plant defense (Figure 6) [50].

In addition to MPK4, the AtWRKY33 protein can also interact with MPK3 and MPK6 (Figure 6) [51]. In *Arabidopsis*, the MPK3/MPK6 activation cascade results in the increased expression of genes related to camalexin biosynthesis and MPK6 and also increases the expression of AtWRKY33. In *atwrky33* mutant plants, functions, such as the expression of genes involved in the production of camalexin through the MPK3/MPK6 cascade and the actual induction of camalexin, are compromised [51]. AtWRKY33 is phosphorylated by MPK3/MPK6 both *in vivo* and *in vitro,* and mutations at the phosphorylation target sites of MPK3/MPK6 in the gene AtWRKY33 are unable to complement the deficiency in the production of camalexin in the loss-of-function mutant *atwrky33*. Possibly by the phosphorylation of MPK3/MPK6, AtWRKY33 leads to the increased expression of AtWRKY33, triggering a positive feedback mechanism that triggers the plant's response to pathogens, including the production of camalexin [51].

In tobacco, the protein NtWRKY1 (representative of the Group I WRKY family) binds to one MAPK known as salicylic acid-induced protein kinase (SIKP) [52]. SIKP is activated after infection with *Tobacco mosaic virus* (TMV) [53] and is also related to HR cell death after induction by an elicitor [54]. SIPK phosphorylates WRKY1, resulting in an increase in the binding activity of this transcription factor to its target DNA sequence, the W–box, which also exists in the tobacco chitinase gene CHN50. In assays for the co-expression of SIPK and WRKY1 in *Nicotiana benthamiana*, cell death by HR is faster compared with plants expressing only SIPK1, suggesting the involvement of WRKY1 in the induction of cell death derived from the HR, which could be a component of the pathway located downstream of SIPK [52]. In *N. benthamiana*, a WRKY that is also a representative of the group I WRKY family, NtWRKY8, is also phosphorylated by SIPK and other MAPKs, specifically the WOUND-INDUCED PROTEIN KINASE (WIPK) and NTF4 (a tobacco mitogen-activated protein kinase related to plant defense response). WRKY8 contains seven potential MAPK phosphorylation sites, five of which are concentrated in the *N*-terminal region. The *N*-terminal region of WRKY8 is characterized by having groups of proline-directed serine residues (SP clusters), which serve as phosphorylation sites for MAPKs *in vitro* and *in vivo*. WRKY8 also contains a D domain adjacent to the *N*-terminus of the SP cluster, which is essential for the effective phosphorylation of WRKY8 in plants. NtWRKY8 phosphorylation increases its binding to W-box sites and also its ability for transactivation. The silencing of WRKY8 decreases the expression of genes related to defense and increases the plant’s susceptibility to pathogens such as *Phytophthora infestans* and *Colletotrichum orbiculare*, demonstrating the importance of this protein in plant defense [55].

WRKY proteins can also interact with proteins involved in autophagy [56,57]. In the nucleus, WRKY33 interacts with ATG18a, an important protein in the autophagy pathway in *Arabidopsis*. The fungus, *B. cinerea* induces autophagic gene expression and the formation of autophagosomes. In plants with *wrky33* loss-of-function, ATG18a induction and the formation of autophagosomes are compromised. Mutants defective for autophagy demonstrate a higher susceptibility to *B. cinerea* and the necrotrophic fungus *Alternaria brassicicola*. The interaction between ATG18a and WRKY33, and consequently with the autophagy pathway, is important for signaling the plant defense response against necrotrophic pathogens [58].

It has been reported that interactions between two or more WRKY proteins are induced by pathogens. The *Arabidopsis* proteins WRKY18, WRKY40 and WRKY60 can form homo- and heterocomplexes; however, the binding activities of these transcription factors vary with the protein region of the complex. Experiments with single loss-of-function mutants for each WRKY protein have demonstrated little change in the phenotype of these mutants for infection by *P. syringae* or *B. cinerea* compared to wild type. Currently, it is known that the double mutants, *wrky18 wrky40* and *wrky18 wrky60*, and the triple mutant, *wrky18 wrky40 wrky60*, are more resistant to *P. syringae* and more susceptible to *B. cinerea* compared to the WT [59]. *atwrky18 atwrky40* mutant plants are highly resistant to the fungus *Golovinomyces orontii*, and WRKY18 and 40 have been shown to act as negative regulators in defense against this fungus [60].

The protein CALMODULIN (CaM) is a modulator of Ca^2+^ signaling in eukaryotic cells [61]. Calmodulin interacts with several proteins, including WRKYs. Through a screen using an *Arabidopsis* library as bait to CaM, the protein AtWRKY17 was identified as an interaction partner of CaM. AtWRKY17 belongs to Group IId of the WRKY family, and its region that binds to CaM is a conserved structural motif (C-motif) that is also found in other representatives of this group [62]. Representatives of the WRKY family Group IId are induced by pathogen infection and also by salicylic acid [63]. The binding site where AtWRKY17 interacts with CaM is commonly found in proteins that are known to interact with CaM [62]. Ten other Group IId WRKY proteins also bind to CaM, and all of their binding domains are similar to the C-motif present in AtWRKY17. Thus, this WRKY/CaM interaction is likely common to all representatives of this group. More studies are needed to establish the role of members of the Group IId family of WRKY transcription factors in signaling mediated by CaM/Ca^2+^ [62].

Transcription factors that belong to the WRKY family may also interact with chromatin remodeling proteins, such as histone deacetylases, which catalyze the removal of acetyl groups on histones. This interaction causes the DNA to become more inaccessible, thereby repressing expression of a gene that is present in this region [64]. *Arabidopsis* AtWRKY38 and AtWRKY62 are part of Group III of the WRKY family. AtWRKY38 and AtWRKY62 appear to have partially redundant functions as negative regulators of basal plant resistance to *P. syringae* and the PR1 gene expression induced by the pathogen [65]. Yeast two-hybrid experiments have identified that HISTONE DEACETYLASE 19 (HDA19) interacts with AtWRKY38 and AtWRKY62, and BiFC assays and co-immunoprecipitations have demonstrated that the interaction occurs in the nucleus and is highly specific. HDA19 expression is also induced by *P. syringae*. HDA19 over-expression in plants results in repression of the transcription activation activities of AtWRKY38 and AtWRKY62 [65].

## 7. NAC Family

In addition to the most studied families of transcription factors involved in defense signaling pathways in plants, such as WRKY and MYB AP2/ERF, factors from other families also participate in modulating responses to biotic stresses. One example is the family of transcription factors containing the NAC domain [66]. The NAC superfamily can be divided into at least seven subfamilies and the functions of NAC genes are defined by their subfamily [66].

**Figure 7:**
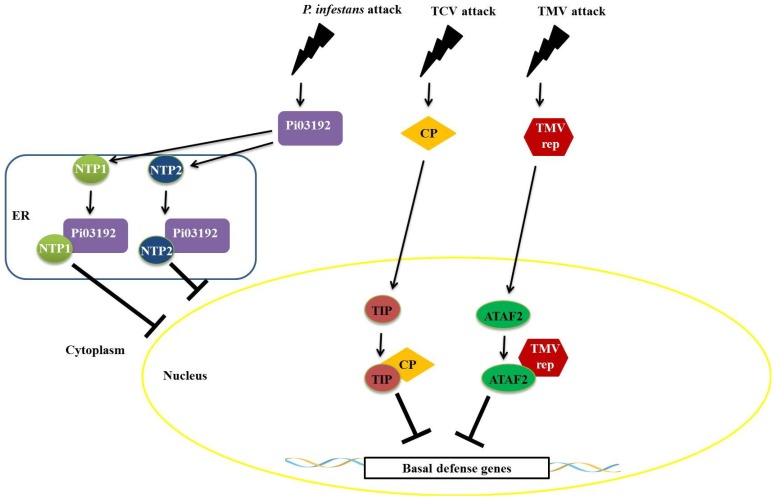
Repression mechanisms of NAC transcription factors mediated by proteins of pathogens. The effector LxLR (Pi03192) of *Phytophthora infestans* interacts with two transcription factors from the no apical meristem (NAM), Arabidopsis transcription activation factor (ATAF), and cup-shaped cotyledon (CUC) (NAC) family (NAC TARGETED BY *PHYTOPHTHORA* 1 and 2 (NTP1 and NTP2)) in the endoplasmic reticulum, thus preventing the localization of these factors to the nucleus. The viral capsid protein from the turnip crinkle virus (TCV) virus binds to TCV-INTERACTING PROTEIN (TIP) factor, repressing the expression of defense genes, favoring systemic infection by plant viruses. The helicase domain of the *Tobacco mosaic virus (*TMV) virus replicase interacts with Arabidopsis transcription activation factor 2 (ATAF2)-suppressing plant defenses.

Recent studies have shown that proteins produced by pathogens interfere with the function of NAC transcription factors. An example is the effector LxLR (Pi03192) produced by *Phytophthora infestans*, which interacts with two transcription factors belonging to the NAC family, termed NAC TARGETED BY *PHYTOPHTHORA* 1 and 2 (NTP1 and NTP2). This interaction occurs in the endoplasmic reticulum and prevents NTP1 localization to the nucleus (Figure 7) [67]. This virus-induced gene silencing (VIGS) of genes encoding these two NAC factors results in increased susceptibility to infection by *P. infestans*, suggesting that these transcription factors play an important role in plant defense [67]. Viral proteins also interact with transcription factors belonging to the NAC family. A NAC protein, designated TCV-INTERACTING PROTEIN (TIP), from *Arabidopsis* interacts specifically with the capsid protein (CP) of turnip crinkle virus (TCV) (Figure 7) [68]. TIP functions through transcriptional activation to promote a basal level of resistance in the plant [68]. The viral CP, produced in infected cells, functions as a virulence factor by binding to TIP to reduce basal resistance and to promote rapid systemic infection (Figure 7). Resistant plants expressing a HYPERSENSITIVE RESPONSE PROTEIN (termed HRT) may guard the TIP protein by detecting a change in TIP caused by the TIP–CP interaction, which will result in a stronger, HR-mediated resistance response [68]. Similarly, an interaction between the helicase domain of TMV 126-/183-kDa replicase protein(s) and the *Arabidopsis* NAC domain transcription factor ATAF2 was identified [69]. In this interaction, TMV suppresses the basal defense pathways during the compatible virus-host interaction with ATAF2 (Figure 7) [68]. This hypothesis is supported by the reduced ability of SA to transcriptionally activate defense-related genes within tissues systemically infected by TMV [69].

NAC proteins interact with protein suppressors of plant defense. In non-induced conditions (without pathogen attack), the protein SUPPRESSOR OF NONEXPRESSOR OF PR GENES INDUCIBLE 1 (SNI1), binds to CBNAC, a calmodulin-regulated NAC transcriptional repressor in *Arabidopsis* [70]. CBNAC binds to the E0-1-1 element of PR1 promoter and SNI1 enhances the DNA-binding activity of CBNAC, consequently enhancing repression of the PR1 gene by SNI1 [70]. In the presence of inducer (during pathogen attack), PR1 gene expression is induced by the translocation of a large amount of active NPR1 to the nucleus and its interaction with TGA transcription factors. The SNI1/CBNAC protein complex can be disassembled by NPR1, calmodulin or other unknown mechanisms [70].

## 8. Conclusions

The evolution of the plant immune response has resulted in a highly effective defense system that is able to resist potential attacks by several types of pathogens. Within this complex defense system are regulatory proteins, such as transcription factors. Over the past few years, a substantial number of proteins that interact with transcription factors involved in plant defenses against pathogens have been identified. In this review, we describe some of the key protein-protein interactions involved in regulating the function of transcription factors important in the defense against biotic stress in plants, such as members of the bZIP families, AP2/ERF, MYB, MYC, WRKY and, more recently, the NAC family. The presence of diversified modular domains involved in direct interactions with different proteins present in transcription factors indicate the diversity of possible interactions, modulating the function of these factors in the process of plant defense.

Various processes of plant defense against pathogen attack are known today, each having a multitude of refined regulatory mechanisms. In this context, examples of interactions are presented, and these interactions can act by modulating the functions of important transcription factors, either by activation or repression of signaling pathways of defense against pathogens from protein-protein interactions (Figure 1, Figure 2, Figure 3, Figure 4, Figure 5 and Figure 6). A broader view of the amazing diversity of the regulatory mechanisms shown during the plant defense reveals the functional redundancy of several transcription factors-interaction partners, such as ANK1 and LSD1 proteins (Figure 1), both genetically unrelated, that interact with transcription factors from the bZIP family, preventing the translocation of these factors to the nucleus. On the other hand, a diverse molecular mode of repression for plant defense pathways is produced by pathogens such as fungi, oomycetes, bacteria and viruses, which suppress the plant response to biotic stress (Figure 3 and Figure 7). We also discuss the key role of the UPS26 system in protein turnover during regulation of the activity of transcription factors in different molecular pathways of plant defense, including the modulation of the concentration of these factors in different subcellular compartments (Figure 1, Figure 3, Figure 4 and Figure 5).

A major question left unanswered about networking of interactions is if those interactions are conserved across plant species, or if they evolved to fine-tune particular responses to specific plant pathogens. The study of *Pseudomonas syringae* (Pst) DC3000 pathogenesis has not only provided several conceptual advances in understanding how a bacterial pathogen employs Type III effectors to suppress plant immune responses and promote disease susceptibility but has also facilitated the discovery of the immune function of stomata and key components of JA signaling in plants [12,27]. The concepts derived from the study of Pst DC3000 provided understanding of pathogenesis mechanisms of other plant pathogens [12]. Similar virulence mechanisms and infection strategies are generally shared in viruses, bacteria, fungi and oomycetes, for example, despite differences in biochemistry, physiology and genetics [12] (Figure 7). In the coming years, it is expected that interacting proteins will be identified by traditional procedures, such as by yeast two-hybrid assays, and by more recently developed methods, such as high density protein microarrays. A particularly important effort will be the integration of knowledge of these complex protein-protein interactions and protein-DNA interactions in the context of the transcription of target genes important for the development of a thorough understanding of the regulatory network of responses to stress caused by pathogens. These studies may lead to a better understanding, not only of the interactions that regulate these transcription factors but also of the important biological processes that these factors modulate.
